# Expression of Concern: Activation of Notch Signaling Is Required for Cholangiocarcinoma Progression and Is Enhanced by Inactivation of p53 *In Vivo*

**DOI:** 10.1371/journal.pone.0226310

**Published:** 2019-12-05

**Authors:** The PLOS ONE Editors

After this article was corrected and republished [1, 2] to address image concerns, additional errors were noted in three panels of Fig 5. Each panel ought to include data for 7 samples, as indicated in the figure labels, but the incorrect number of lanes were presented in the following:

- Fig 5A: The Actin panel for the Snail and E-cadherin experiments has 8 lanes instead of 7.
- Fig 5B: The Actin panel for the Snail and E-cadherin experiments has 8 lanes instead of 7.
- Fig 5B: The N-cadherin panel and corresponding Actin panel each have 6 lanes instead of 7.

The authors explained that these images were cropped incorrectly when the original figure was composed such that they included control lanes (Actin panels for Snail and E-cadherin, Fig 5A and 5B) or excluded DMSO lanes (N-cadherin and corresponding Actin panels, Fig 5B). Please see the updated Fig 5 in which these issues are addressed here. Note, the original images underlying Fig 5 are no longer available except for those included in the prior Correction [2].

The underlying data supporting Fig 4 of the article [1] are provided here in S1 and S2 Files. The underlying data supporting other results in this article are not available.

In light of the cumulative concerns about this article and the unavailability of supporting data, the *PLOS ONE* Editors issue this Expression of Concern.

For reference, the original version of the article is included here in S3 File. Note that the original version of Fig 3A included an image that was not licensed for reproduction and distribution under the terms of the Creative Commons Attribution License. When the previous Correction [2] was posted, the article was republished to remove the previously published content from the article. The image in question is redacted in S3 File.

The work reported in this article [1] was completed when the corresponding author (RRP) was affiliated with Department of Internal Medicine I, Medical University Hospital, Tuebingen, Germany, as is noted on the published article. RRP is currently affiliated with Department of Internal Medicine II, Gesundheit Nord Klinikverbund Bremen.

**Fig 5:**
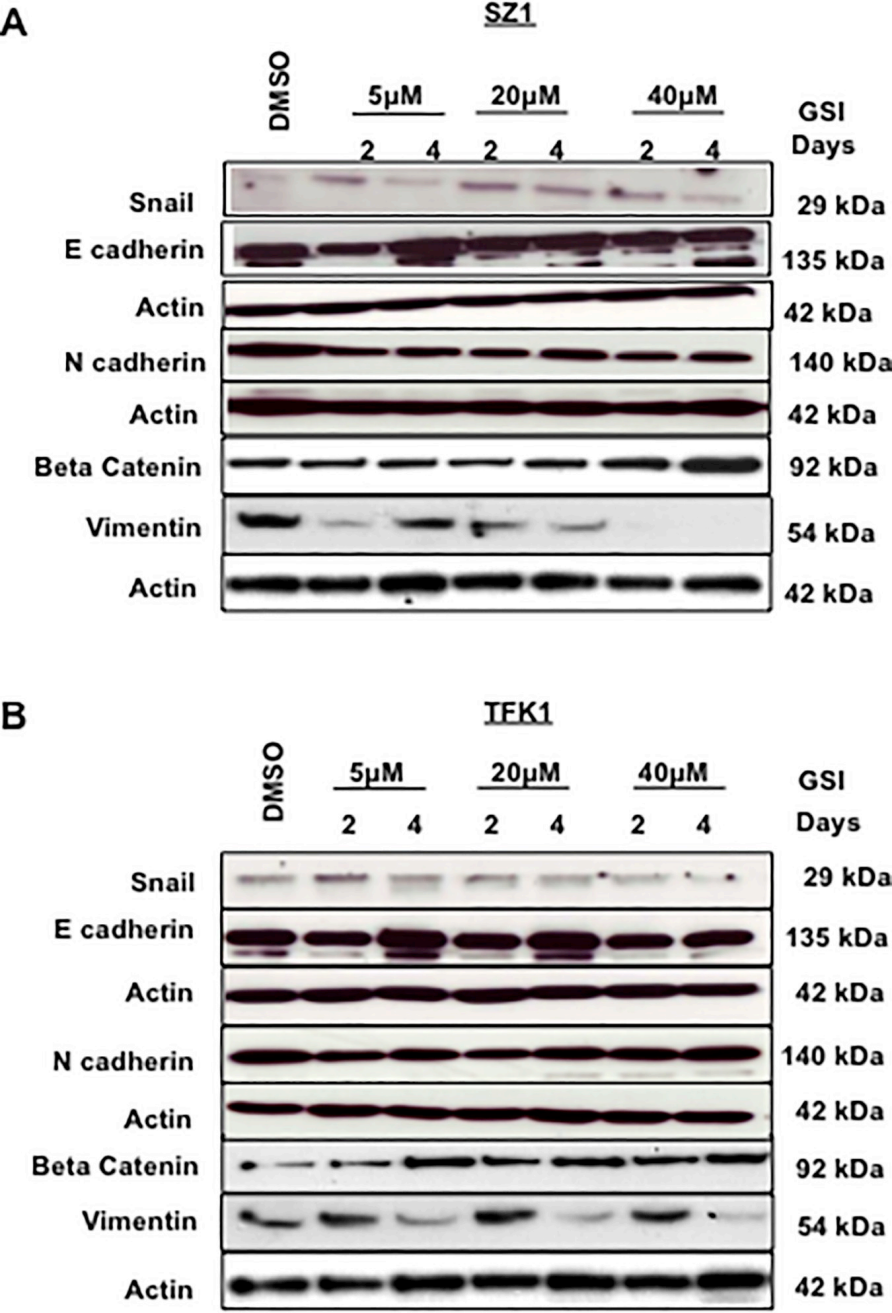
Change in the expression of epithelial and mesenchymal cell markers after GSI IX treatment in human cholangiocarcinoma. (A) SZ1 and (B) TFK1 cells were treated with control (DMSO) and GSI (5 μM, 20 μM and 40 μM) for 48 h and 96 h. The expression of EMT markers: E-cadherin, N-cadherin, Snail, Beta Catenin and Vimentin were analyzed by Western blot. β-actin was used as a loading control. Both A) SZ1 and (B) TFK1 cells showed an increase of epithelial marker E-cadherin, Beta Catenin and a decrease of Snail and Vimentin. However, N-cadherin expression was unchanged for both cholangiocarcinoma cell lines.

## Supporting information

S1 File Underlying data for quantitative results shown in Fig 4A. (XLS)

S2 File Underlying data for quantitative results shown in Fig 4B. (XLS)

S3 File Original article PDF with copyrighted figure panel redacted. (PDF)
