# Supplementary material for: Expression of Concern: Activation of Notch Signaling Is Required for Cholangiocarcinoma Progression and Is Enhanced by Inactivation of p53 In Vivo
Source: PLoS One. 2019 Dec 5;14(12):e0226310. doi: 10.1371/journal.pone.0226310 (PMC6894761; doi:10.1371/journal.pone.0226310)
Supplement: S3 File — (PDF) [file pone.0226310.s003.PDF]

PLOS | ONE

Activation of Notch Signaling Is Required for Cholangiocarcinoma Progression and Is Enhanced by Inactivation of p53 *In Vivo*

Mona El Khatib[1], Przemyslaw Bozko[1], Vindhya Palagani[1], Nisar P. Malek[1], Ludwig Wilkens[2], Ruben R. Plentz[1]*

1 Department of Internal Medicine I, Medical University Hospital, Tuebingen, Germany, **2** Institute of Pathology, Nordstadt Krankenhaus, Hannover, Germany

Abstract

Cholangiocacinoma (CC) is a cancer disease with rising incidence. Notch signaling has been shown to be deregulated in many cancers. However, the role of this signaling pathway in the carcinogenesis of CC is still not fully explored. In this study, we investigated the effects of Notch inhibition by γ-secretase inhibitor IX (GSI IX) in cultured human CC cell lines and we established a transgenic mouse model with liver specific expression of the intracellular domain of Notch (Notch-ICD) and inactivation of tumor suppressor p53. GSI IX treatment effectively impaired cell proliferation, migration, invasion, epithelial to mesenchymal transition and growth of softagar colonies. *In vivo* overexpression of Notch-ICD together with an inactivation of p53 significantly increased tumor burden and showed CC characteristics. *Conclusion*: Our study highlights the importance of Notch signaling in the tumorigenesis of CC and demonstrates that additional inactivation of p53 *in vivo*.

Citation: El Khatib M, Bozko P, Palagani V, Malek NP, Wilkens L, et al. (2013) Activation of Notch Signaling Is Required for Cholangiocarcinoma Progression and Is Enhanced by Inactivation of p53 *In Vivo*. PLoS ONE 8(10): e77433. doi:10.1371/journal.pone.0077433

Editor: Peiwen Fei, University of Hawaii Cancer Center, United States of America

Received June 10, 2013; **Accepted** September 2, 2013; **Published** October 30, 2013

Copyright: © 2013 El Khatib et al. This is an open-access article distributed under the terms of the Creative Commons Attribution License, which permits unrestricted use, distribution, and reproduction in any medium, provided the original author and source are credited.

Funding: Funding was provided by German Research Foundation (PL 468/4-1). The funders had no role in study design, data collection and analysis, decision to publish, or preparation of the manuscript.

Competing Interests: The authors have declared that no competing interests exist.

* E-mail: Ruben.Plentz@med.uni-tuebingen.de

These authors contributed equally to this work.

Introduction

Cholangiocarcinoma (CC) is a cancer disease which is increasing worldwide [1,2]. Representing the second most common primary hepatobiliary cancer shows the need for a better understanding of the tumor development [3]. Most of the CC tumors are adenocarcinomas arising from epithelial cells lining the intra- and extrahepatic biliary tract system [4,5]. Known risk factors are primary sclerosing cholangitis (PSC), cirrhosis, chronic viral hepatitis B and C infection, diabetes, obesity, smoking, alcohol intake and toxin exposure like Thorotrast and Dioxins [6–8]. Often CC is detected at an advanced stage and patients show up with an extension of the disease which impairs the possibility of curative surgery. Thus, treatment by photodynamic therapy (PDT), systemic chemotherapy and/or radiotherapy are the only options for patients with inoperable disease [9–11]. Different studies have shown that CCs are characterized by a series of highly recurrent genetic abnormalities, including KRAS, BRAF, p53, SMAD and p16^{INK4a} mutations [12–17]. Currently, the combination of Gemcitabine and Cisplatin is the standard chemotherapeutic regimen for patients undergoing first line treatment [18,19]. However, standard chemotherapies only offer limited benefit and new strategies are still needed to overcome this deadly disease.

The Notch signalling pathway is involved in the development and progression of several malignancies [20]. The interaction of Notch ligands with their receptors, promotes a γ-secretase-dependent cleavage of the Notch receptor and release of the Notch intracellular domain (NICD) resulting in activation of the pathway [20,21]. NICD translocates to the nucleus and induces target genes like Hairy enhancer of split (Hes1). Upregulation of the Notch1 receptor by cholangiocytes in PSC and CC has been described by Ishiumura and colleagues [22]. Furthermore, losses of the Notch ligand Jagged1 or the Notch 2 gene result in congenital hypoplasia of the biliary system, the Alagille syndrome [23–25]. Analysis of mice after liver-specific inactivation of RBP-Jκ, a common transcriptional mediator of Notch signaling, revealed a reduced number of biliary cells differentiating from hepatoblasts [26]. The first clinical observation that aberrant expression of Notch1 and 3 correlates with poor survival in extrahepatic CC were made by Yoon et al. [27]. Recently, it was demonstrated that intrahepatic CCs can orginate from hepatocytes and that activation of Notch and AKT can convert hepatocytes into intrahepatic CCs [28,29]. It was also reported that liver-specific expression of the intracellular domain of Notch2 in mice caused biliary hyperplasia [30]. Zhou et al. reported that Notch1 might be involved in carcinogenesis of intrahepatic cholangiocarcinoma [31]. Data from our own group underlined the important role of Notch signaling in CC and identified cyclin E as a target of Notch [32]. Epithelial mesenchymal transition (EMT) which can promote cancer invasion and metastasis [33,34] and the role of Notch signaling in CC carcinogenesis remain not fully discovered and the interaction of the tumor suppressor gene p53 and NICD expression not investigated. In the present study, we sought to further examine the potential benefit of targeting Notch in CC by studying in detail the impact of γ-secretase

Table 1. Summary of observation in NotchICD :: AlbCre :: p53 L/L mice.

ID	Gender	Age	Macroscopic observations	Microscopic observations
8K	F	3 month	no tumor	no carcinoma cells
12K	F	3 month	no tumor	no carcinoma cells
41H	M	6 month	no tumor	no carcinoma cells
42H	M	6 month	no tumor	no carcinoma cells
34I	M	6 month	small tumor	carcinoma cells
21I	F	6 month	tumor with liver metastases	carcinoma cells
5H	F	8 month	tumor	carcinoma cells
32G	M	9 month	tumor with liver metastases	carcinoma cells
43H	M	9 month	tumor with liver metastases	carcinoma cells
44H	M	9 month	tumor with liver metastases	carcinoma cells

Macroscopic implies visible tumor and microscopic means no visible tumor by eyes.
doi:10.1371/journal.pone.0077433.t001

inhibitor IX (GSI) in human CC cell lines and by establishing a transgenic mouse model with liver specific expression of the intracellular domain of Notch (Notch-ICD) and inactivation of p53.

Our work reveals that GSI can impair cell proliferation, EMT, migration and invasion in human CC cell lines. Notch activation and loss of p53 together is showing a increased tumor burden *in vivo*, showing the underlying role of Notch in CC pathogenesis. Our findings support the development of therapeutic strategies targeting Notch signaling in human CC.

Materials and Methods

Cell Culture

The human cholangiocarcinoma cell lines TFK1, SZ1, and EGI1 were generously provided by Prof. Nisar Malek. TFK1 and EGI1 cells were originally obtained from the DSMZ (German Collection of Microorganisms and Cell Cultures, Human and Animal Cell Lines, Braunschweig, Germany). SZ-1 was established from a surgically resected tumor specimen (from a patient with CC), which was histologically diagnosed as adenocarcinoma of moderate differentiation with cholangiolar differentiation [32]. All cell lines were cultured in RPMI 1640+ Glutamax (Invitrogen, Karlsruhe, Germany) supplemented with 10% FCS (Biochrom, Berlin, Germany) and 100 U/ml penicillin/streptomycin (Invitrogen, Karlsruhe, Germany) at 37°C in 5% CO_2.

Drugs and Treatment

γ-secretase inhibitor IX (GSI) (Calbiochem, EMD Chemicals Inc., Darmstadt) was prepared as a 10 mM stock in dimethyl sulfoxide, DMSO (AppliChem, Darmstadt, Germany). Cells were treated with DMSO or γ-Secretase inhibitor IX (5 µM, 20 µM, 40 µM) and were analyzed after 24, 48 and 96 hrs.

Proliferation Assay

In order to measure the effect of Cyclopamine on cell proliferation, cells were plated at a concentration of 1×10^4 cells/ml in a 96 well plate overnight. Then cells were treated with DMSO, NS1, Tomatidine,different concentrations of Cyclopamine (5, 10 and15 µM) and 5E1 (40 µg/ml) for different time points (1–4 days). At the respective time points, 10 µL WST-1 reagents (Roche Diagnostics, Mannheim) was added to each well and incubated for 2 h at 37°C. The absorbance was detected at a wavelength of 492 nm with reference wavelength of 650 nm.

Invasion Assay

Cells (2.5×10^5 cells/2 ml) were seeded in serum free media into each well of the 6-well BD BioCoat^{TM} Matrigel^{TM} Invasion Chamber (BD Biosciences, Bedford, UK). The cells in the inserts were simultaneously treated with Cylopamine (5, 10 and 15 µM), E-caderin siRNA (100 nM) and the controls (DMSO, Tomatidin and control siRNA-A). The inserts were placed into the BD Falcon TC Companion Plate containing 10% FCS and incubated for 48 h hours in a humidified tissue culture incubator, at 37°C, 5% CO_2 atmosphere. Then the invading cells were fixed with 100% methanol and stained with 1% toluidine blue in 1% borax. Cell were then counted under the microscope (Leica DM 5000 B, Leica, Wetzlar). The calculation of the invading cells were done according to the BD protocol

Table 2. Summary of observation in NotchICD :: AlbCre mice.

ID	Gender	Age	Macroscopic observations	Microscopic observations
2G	F	6 month	no tumor	no carcinoma cells
10G	F	6 month	no tumor	no carcinoma cells
5H	M	6 month	no tumor	no carcinoma cells
14H	M	6 month	no tumor	no carcinoma cells
17I	M	6 month	no tumor	no carcinoma cells

Macroscopic implies visible tumor and microscopic means no visible tumor by eyes.
doi:10.1371/journal.pone.0077433.t002

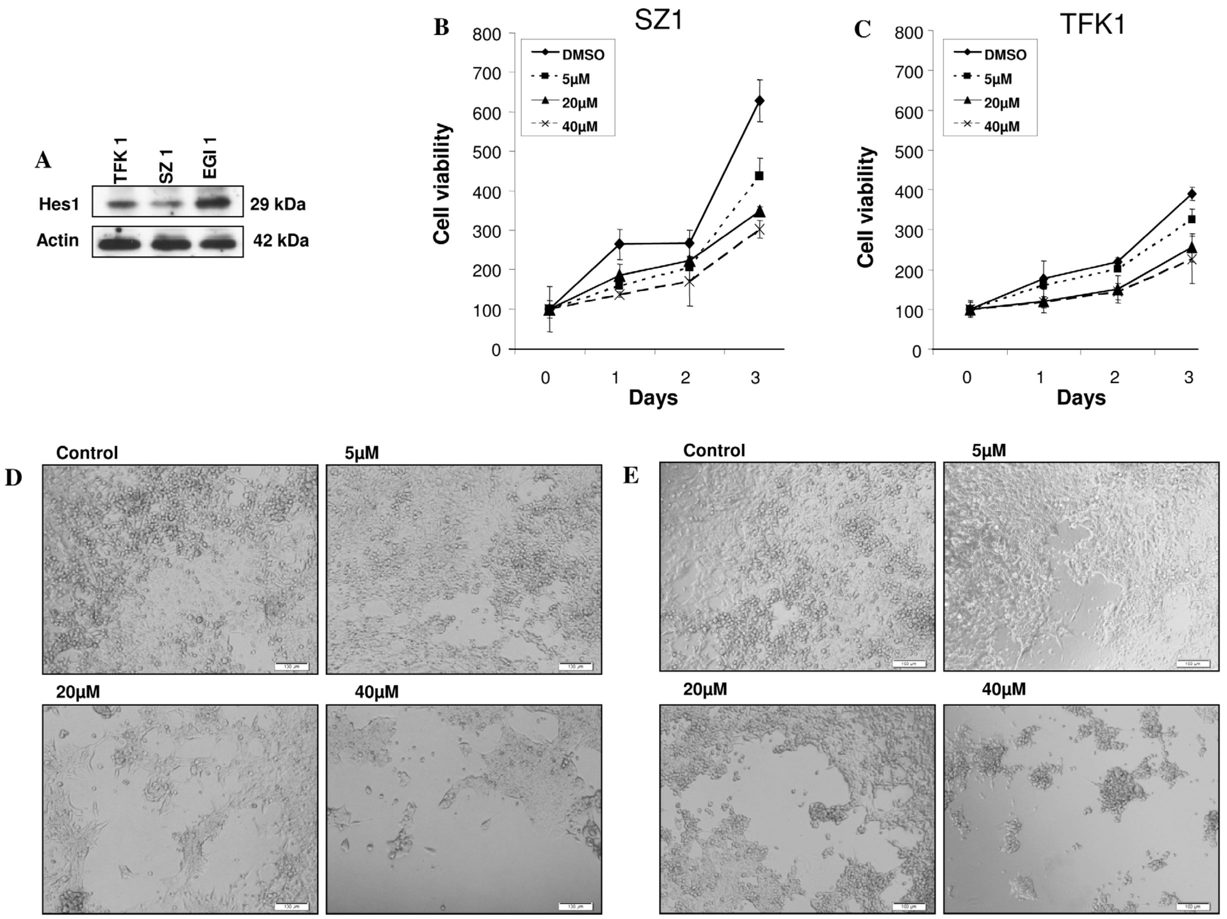

Figure 1. GSI IX inhibits cell proliferation in human cholangiocarcinoma cell lines. TFK1, SZ1 and EGI1 cells showed a different expression of the Notch downstream target Hes1 (A). The cell proliferation of (B) SZ1 and (C) TFK1 cells was measured by cell proliferation assay, GSI (5 µM, 20 µM, 40 µM) inhibited cell proliferation in a dose- and time-dependent manner. Light microscopic pictures (10X magnification) were taken at 96 h to show the effect of GSI on cell proliferation of (D) SZ1 and (E) TFK1. Note that these results reveal the anti-proliferative effects of GSI IX on human cholangiocarcinoma cells.
doi:10.1371/journal.pone.0077433.g001

where

$$\text{Invasion Index} = \frac{\%\,\text{Invasion Test Cell}}{\%\,\text{Invasion Control Cell}}$$

Migration Assay

Cholangiocarcinoma cell lines (SZ1 and TFK1) were seeded in a 6-well plate and left to reach 80% confluency. Initially, cells were starved for 24 h in media containing 2% FCS. Then SZ1 and TFK1 were further incubated for 48 h in the starvation media containing either the controls (DMSO, Tomatidin, control siRNA-A, Cyclopamine or E-cadherin siRNA). Afterwards a scratch was done using a white tip for each treatment. Then cells were washed with PBS and photographed using Leica DMI 6000 B microscope (Leica, Wetzlar). Cells were incubated for an additional 24 h after which the photographs were taken for the wounded area. The migrating cells were calculated according to the following formula:

$$\text{Migration Index} = \frac{\text{Width of the wound at 0 h} - \text{Width of the wound at 24 h}}{\text{Width of the wound at time 0 h}} \times 100$$

Annexin V Apoptosis Assay

Cell death was detected by the use of FITC Annexin V Apoptosis Detection Kit II (BD Biosciences, San Diego, USA). Cells were cultured in 6-well plates at a plating density of 8×10^4cells/ml after which they were treated with the control (DMSO) or GSI for 48 or 96 hrs. Cells were collected and stained with annexin V. The signal was detected using FACS calibur flow cytometer (BD, Heidelberg) and analyzed using FlowJo Version 8.7 software (Tree Star Inc., Ashland, USA).

Measuring of DNA Fragmentation/subG1 Population by Propidium Iodide Staining Following Flow Cytometry Analysis

DNA fragmentation/subG1 population was determined by flow cytometry using an FACS calibur flow cytometer (BD, Heidelberg)

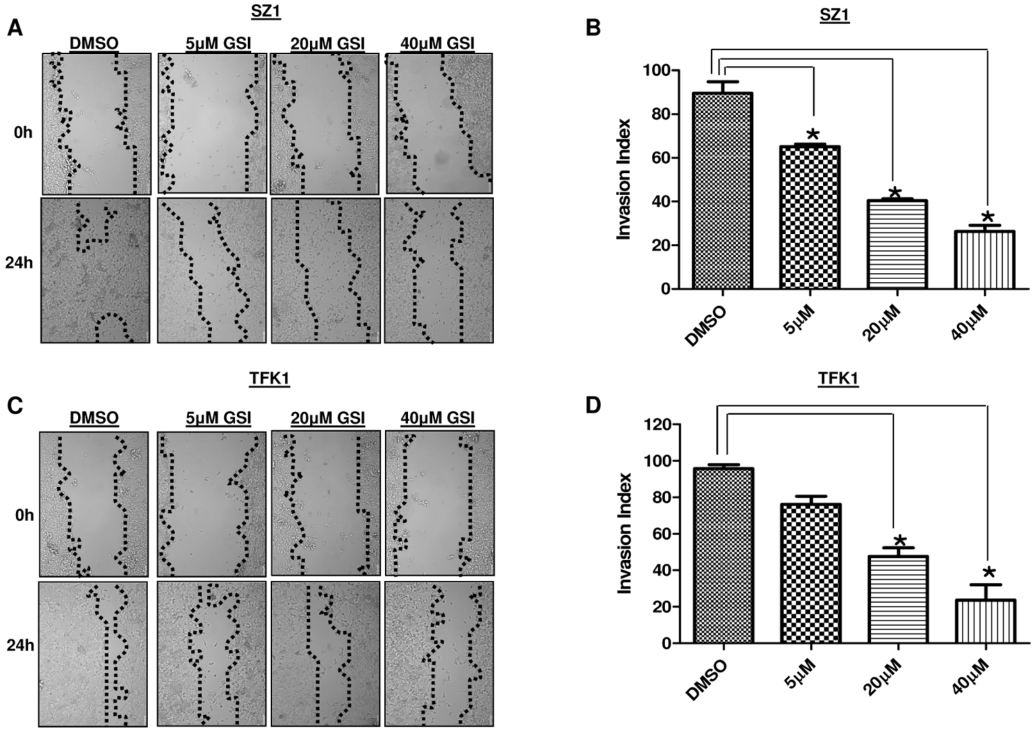

Figure 2. Notch plays a pivotal role for the regulation of migration in human cholangiocarcinoma cells. Treatment with GSI IX suppresses the migration potential of human cholangiocarcinoma cell lines SZ1 and TFK1. Wound healing experiments of (A) SZ1 and (C) TFK cells cultured with GSI (5 μM, 20 μM, 40 μM) or control (DMSO). A scratch was made at (time 0 h) in both SZ1 and TFK1 and maintained for 24 h in conditioned medium with GSI or DMSO. The dotted lines are representing the edges of the wound. Photographs were taken under light microscope (10X magnification). After 24 h (A) SZ1 showed significant inhibition under 5–40 μM GSI and (C) TFK1 with a dose of 20–40 μM GSI treatment. In DMSO treated cells 80% to 90% of the wound healing was observed after 24 hrs. (A,C) The migration index (B,D) was calculated as described in Material and Methods and plotted in bar graphs. P values were calculated with ANOVA analysis of variance along with Bonferroni post test. The error bar represents standard deviation. Differences were considered as statistically significant (*) when the P-value was less <0.05.

doi:10.1371/journal.pone.0077433.g002

Content Removed

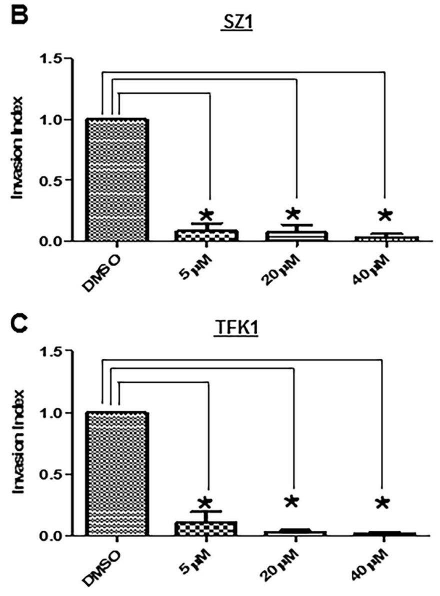

Figure 3. GSI IX attenuate invasion of human cholangiocarcinoma cells. SZ1 (A) and TFK1 (A) cell lines were treated for 48 h with control (DMSO) and GSI (5 μM, 20 μM, 40 μM) to investigate the effect of GSI on invasiveness of human cholangiocarcinoma cell lines. The number of cells that invaded through the membrane was determined by light microscope (20X magnification) counterstained and invasion index (B,C) was calculated as described in Material and Methods and plotted in bar graphs. Both TFK1 and SZ1 showed significant decrease in number of invading cells by light microscope. P values were calculated with ANOVA analysis of variance along with Bonferroni post test. The error bar represents standard deviation. Differences were considered as statistically significant (*) when the P-value was less <0.05.

doi:10.1371/journal.pone.0077433.g003

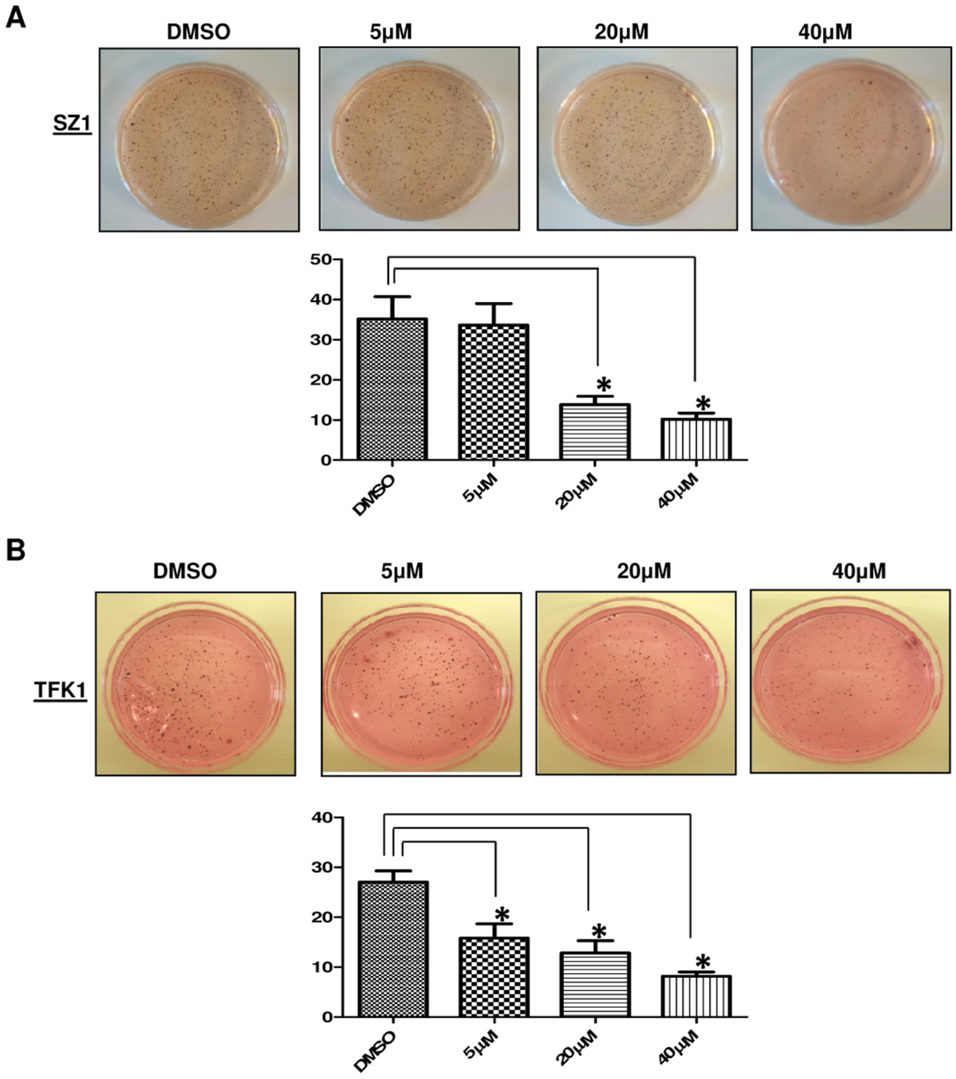

Figure 4. GSI IX treatment significantly inhibited the colony formation ability of cholangiocarcinoma cell lines. (A) Soft agar assay of GSI-treated human cholangiocarcinoma cell line (SZ1), with quantification. Compared to DMSO (control) GSI inhibits colony formation at a concentration of 20 μM. (B) Soft agar assay of GSI-treated human cholangiocarcinoma cell line (TFK1), with quantification. Compared to DMSO (control) GSI inhibits colony formation at a concentration of 5 μM. P values were calculated with ANOVA analysis of variance along with Bonferroni post test. The error bar represents standard deviation. Differences were considered as statistically significant (*) when the P-value was less <0.05.
doi:10.1371/journal.pone.0077433.g004

equipped with an argon laser to give 488 nm light. The cells were fixed in 70% ethanol at −20C, rehydrated in PBS, and stained with PBS containing PI (10 μg/mL) and RNase A (50 μg/mL) for 30 minutes at room temperature. The percentage of cells in sub G1 phase of the cell cycle was calculated by FlowJo Version 8.7 software (Tree Star Inc., Ashland, USA).

Immunohistochemistry

Tissue sections were fixed in 4% formalin (Sigma-Aldrich, St. Louis, USA) overnight, stored in PBS and embedded in paraffin. For immunohistochemistry slides were deparaffinized using an oven (GE Healthcare Limited, Buckinghamshire, UK) and rehydrated in decreasing ethanol concentrations (Merck, Darmstadt). Antigen retrieval was performed by heating the slides in pressure cooker with Antigen Unmasking Solution (Vector Laboratories, Inc., Burlingame). The slides were then washed in PBS and incubated for 10 min in 1% H2O2 (Sigma-Aldrich, St. Louis, USA), rinsed with PBS, and incubated 1 h in blocking solution (5% normal serum 0.3% Triton X-100, Vector Laboratories, Inc., Burlingame). Hybridization with the primary antibody E-cadherin (Cell signaling, 24E10), N-cadherin (Millipore, EPR1792Y) and Cytokeratin 7 (CK7, DAKO, Germany) was carried out overnight at 4°C. Then secondary antibody (1:200,Vector Laboratories, Inc., Burlingame) was incubated for 1 h at room temperature. The manufacturer's protocols were used for ABC and DAB substrates (Vector Laboratories, Inc., Burlingame). Slides were counterstained with hematoxylin and dehydrated in 40%, 70%, 90%, and 100% ethanol. Finally, slides were cleared with Rhotihistol (Roth, Karlsruhe) and mounted with Permount Toluene Solution (Fisher Scientific, New Jersey, USA).

Protein Extraction and Western Blotting

SZ1 and TFK1 cells cultured with GSI treatment for immunoblots were collected and rinsed with cold phosphate-buffered saline (PBS). Then harvested cells were lysed in lysis buffer containing 20 mM Tris, 150 mM NaCl, 1 mM EDTA,

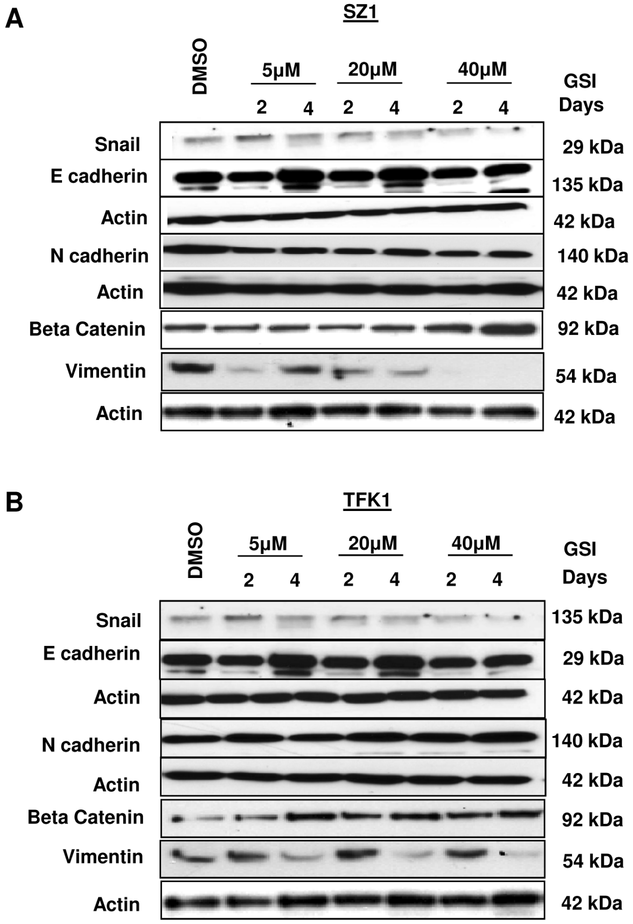

Figure 5. Change in the expression of epithelial and mesenchymal cell markers after GSI IX treatment in human cholangiocarcinoma. (A) SZ1 and (B) TFK1 cells were treated with control (DMSO) and GSI (5 μM, 20 μM and 40 μM) for 48 h and 96 h. The expression of EMT markers: E-cadherin, N-cadherin, Snail, Beta Catenin and Vimentin were analyzed by Western blot. β-actin was used as a loading control. Both A) SZ1 and (B) TFK1 cells showed an increase of epithelial marker E-cadherin, Beta Catenin and an decrease of Snail and Vimentin. However, N-cadherin expression was unchanged for both cholangiocarcinoma cell lines.
doi:10.1371/journal.pone.0077433.g005

1 mM EGTA, 1% Triton X-100 and protease and phosphatase inhibitor (Protease Inhibitor Cocktail Tablets, Roche, Mannheim). The concentration of extracted protein was determined using DC protein assay kit (Biorad, München) following manufacturer's instruction. The absorption was measured at 650–750 nm using a microplate reader (Titertek-Berthold, Pforzheim). For immune blotting the cell lysates were loaded at a protein concentration of 30 μg per well. Gel electrophoreses (12% acrylamide gels) was performed (Biorad, München). The membranes were blocked using 5% dried milk (AppliChem, Darmstadt, Germany) for 30 minutes at room temperature. Then they were probed with primary antibodies against Hes1 (1:10.000; Toray Industries Inc., Kamakura, Japan), E-cadherin (1:1000; Cell signaling, 24E10), N-cadherin (1:10.000; Millipore, EPR1792Y), Snail (1:1000; Cell signalling), Beta Catenin (1:1000; Cell signalling), Vimentin (1:1000; Cell signalling) and Actin (1:10.000; Sigma, AC 74), the signal was Detected by AmershamHyperfilm ECL (GE Healthcare Limited, Buckinghamshire, UK).

Animals

ROSA26 NICD mice [35] were crossed with AlbCre and $p53^{lox/lox}$ mice [36] to generate mice with a liver specific over expression of the activated form of the Notch1 receptor and inactivation of the tumor suppressor gene p53. In total n = 10 mice were analyzed and killed at 3, 6, 8 and 9 month. In addition, n = 5 control mice with AlbCre and NICD expression only [32] were dissected at 6 month. Health status were inspected every second day. Pictures were taken from the liver or visible tumors and tissues were fixed in 4% formalin for histology. Animal data are summarized in Table 1 & 2.

Ethics Statement

The mice used in this study were maintained in the animal care of Medizinischen Hochschule Hannover (MHH) facility. All experimental protocols were reviewed and approved by institutional guidelines for animal care of MHH and lower Saxony (protocol no: AZ: 10/088), and all studies were performed according to the methods approved in the protocol. Use of human tissue was reviewed by local ethic committee of MHH (protocol no: SZ 11/203) and written consent form from the donor was obtained. MHH approved the usage of human tissue for the underlying study.

Statistical Analysis

All the experiments were repeated 2–3 times. The results were analyzed using software Graphpad prism version 5.0 (GraphPad Software, San Diego, CA, USA) and SPSS Version 11.0 (SPSS, Chicago, USA). The tests include one way ANNOVA analysis of variance and students *t*-test along with Bonferroni post test and paired and unpaired t-tests. Differences were considered as statistically significant when the P-value was less <0.05.

Results

Notch Signalling Pathway is Essential for the Maintenance of the Proliferation and Growth of Human Cholangiocarcinoma Cells

To determine activation of Notch signaling in human cholangiocarcinoma cell lines, expression of the Notch downstream target Hes1 was assessed in TFK1, EGI1 and SZ1 by western blotting (Fig. 1A). Next, the effect of GSI IX on the proliferation of three human cholangiocarcinoma cell lines was determined by MTT assay. As illustrated in Fig. 1 and Fig. S1, GSI treatment reduced the number of viable TFK1, SZ1, EGI 1 in a dose and time dependent manner (Fig. 1B–E, Fig. S1). Phase contrast images are supporting the cell proliferation results (Fig. 1D,E). These results confirm that Notch signaling is necessary for the proliferation of cholangiocarcinoma cells and that targeting Notch might be a therapeutic approach.

Treatment by γ-secretase Inhibitor IX Results in an Inhibition of Human Cholangiocarcinoma Cell Migration, Invasion and Colony Formation

Notch signalling has been implicated in the invasive growth of a number of cancer types. The role of Notch in CC *in vitro* carcinogenesis was not fully studied yet. Thus, we sought to explore the impact of GSI IX on the human CC cell lines TFK1 and SZ1. First, we examined the migration ability by employing wound healing migration assays (Fig. 2A–D). Twenty four hours after the scratch, cell migration was significantly ($P<0.05$) inhibited at and above 20 μM GSI IX in both cell lines (Fig. 2 A–D). Only TFK1 cells showed already with 5 μM GSI a

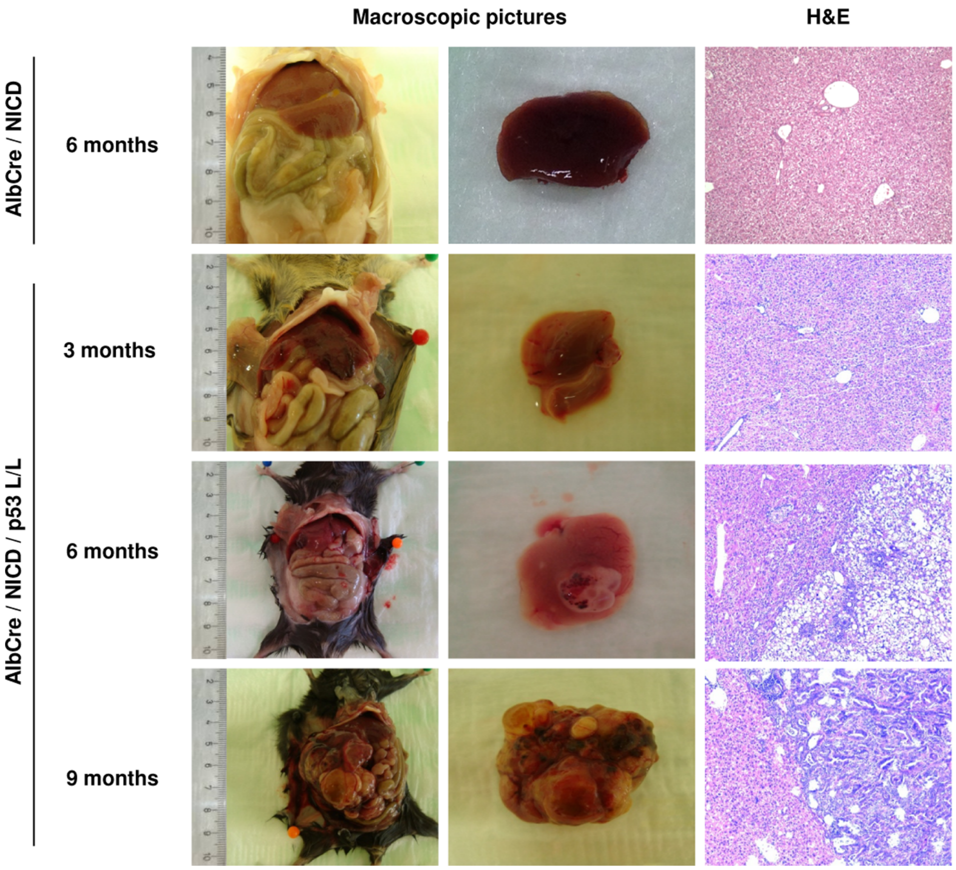

Figure 6. NICD overexpression and loss of p53 is influencing the tumor development of cholangiocarcinomas in mice. Macro- und microscopic pictures of mice with expression of AlbCre and Notch-ICD and inactivation of p53 at different timepoints like indicated. Each mouse was dissected at the indicated timepoint. H&E staining (20X magnification) was performed and analysis was accordingly done in collaboration with an independent pathologist. Note, there was no tumor either macroscopically or microscopically detected with an age of 3 month. Starting with an age of 6 month, mice with expression of AlbCre, Notch-ICD and loss of p53 developed cholangiocarcinomas.
doi:10.1371/journal.pone.0077433.g006

significant impairment of cell migration (Fig. 2 C,D). In contrast, 80–90% wound healing was seen after 24 h in all untreated cells. Next, we tested cell invasion with transwell chambers (Fig. 3 A–C). CC cells were plated in wells of an invasion chamber in the presence of different drug concentrations (5 μM, 20 μM, 40 μM) and experiments were conducted as described in Material and Methods. As shown in Fig. 3 A–C cell GSI IX inhibited cell invasion in a concentration dependent manner. Reduction of up to 90% in the number of invading cells compared to the control group was seen. Even the lowest concentration with GSI IX (5 μM) significantly inhibited cell invasion in TFK1 and SZ1 cells. Furthermore, GSI IX strongly inhibited the ability of human CC cells to form clones in soft agar compared with DMSO treated cells, indicating that active Notch signaling is necessary for anchorage-independent clonogenic growth of these cells (Fig. 4. A,B). Additionally, to analyze apoptosis we performed Annexin V assay. GSI IX treated cells showed a increase of apoptotic cells after 96 hours (Fig. S2A). We also demonstrated an increase in SubG1 population at 96 hours without any significant changes between the different GSI IX dosages the highest percentage being at 96 h; 39% of SZ1 and 28% of TFK1 cells in the subG1 fraction of the cell cycle (Fig. S2B). Thus, treatment with GSI IX can successfully inhibit migration, invasion and colony formation of human CC cells and induces levels of apoptosis, which is correlated with the increase in the population of cells in the SubG1 fraction of the cell cycle.

Notch Signalling Pathway Regulates Epithelial Mesenchymal Transition in Human Cholangiocarcinoma Cell Lines

The epithelial-to-mesenchymal transition (EMT) is a process during which cells undergo a developmental switch from an epithelial phenotype to a mesenchymal phenotype [33,34]. The EMT phenotype has been described to contribute to many GI cancer invasions, but also treatment resistance in cancer cells. We hypothesized that blocking the Notch signaling pathway can partially reverse EMT in CC. To confirm that GSI IX treatment influences EMT we have checked the protein expression of epithelial and mesenchymal markers after different exposure to GSI IX (5 μM, 20 μM, 40 μ) treatment and different time points (2 days, 4 days). We observed a time and dose dependent increase in the protein expression of E-cadherin and Beta-Catenin after GSI IX treatment compared to the control (Fig. 5 A,B). Zinc-finger protein Snail and Vimentin showed decreased expression especially after 4 days treatment with GSI (Fig. 5 A,B). However, there was no change in the expression of N-cadherin. In addition, we verified the activation E- and N-cadherin by checking their expression in human CC tissues by immunohistochemistry staining (Fig. S3).

NICD Overexpression together with Loss of p53 is Enhancing Tumor Development (Growth) *in vivo*

We hypothesized that the overexpression of the activated form of the Notch1 receptor together with an inactivation of the tumor suppressor gene p53 will enhance tumor development. To directly proof our hypothesis we crossed transgenic mice with specific overexpression of the intracellular domain of Notch 1 (Rosa26-Notch1IC) to a mouse line which expresses cre-recombinase under the control of the albumin regulatory elements and the alpha-fetoprotein-enhancers (AlbCre), as well with loxed p53 ($p53^{lox/lox}$) [35,36].

Data from our group showed that mice with only NICD expression in the liver develop with an age of 7 month areas with small cells with an epithelial appearance [32]. As early as 8 month after birth changes of nuclear morphology, but no cancer was reported. In our study, mice with AlbCre and NICD showed neither macro−, nor microscopically abnormalities (Fig. 6, Table 2). Mice with additional loxed p53 ($p53^{lox/lox}$) showed with an age of 3 month also no important changes (Fig. 6, Table 1). However, 6 month after birth in two animals out of four, we observed a macroscopic detectable tumor in the liver (Fig. 6, Table 1). Histological analysis revealed carcinoma cells growing in a pattern with abortive glandular pattern. The tumor cells revealed moderate to high pleomorphism of the nuclei with occurring of atypic mitoses. The tumor cells are accompanied by dense fibrous tissue with some inflammatory cells. In remaining portal triads there were also changes of the cholangiolar structures in particular for those seen in the ductal plates (Fig. 6). All investigated mice with an age between 8 and 9 month ($n = 4$) (Table 1, Figure 6) developed big tumors of the liver, but no metastases. Histological analysis confirmed tumor growth pattern as described before (Fig. 6). Immunohistochemical staining using CK7 confirmed the epithelial lineage of the tumor cells (Fig. S4). Worth mentioning, all animals including the older ones did not show any signs of discomfort during their observation periods. Our data confirm the leading role of the Notch signaling pathway in the development of CC and the impact of inactivation of p53 in this connection.

Discussion

Several studies have implicated the involvement of Notch in the initiation and progression of many different haematological malignancies and solid tumors. The role of Notch for CC carcinogenesis was not described in detail yet. In the present study, we firstly confirmed that the Notch target gene Hes1 is expressed in different human CC cell lines, including one primary human CC cell line. Treatment by γ-secretase inhibitor IX (GSI) induced dose- and time-dependent growth inhibition in two human CC cell lines (TFK1, SZ1), as indicated by WST assay.

Epithelial to mesenchymal transition (EMT) is a physiological process that allows morphological and genetic changes of cancer cells, which is the basis for high metastatic potential [37,38]. E-cadherin is known to be expressed by liver progenitors and biliary epithelial cells [39]. There are only a few reports about EMT in CC. Araki et al. demonstrated for extrahepatic CCC that the cadherin switch promotes tumor progression via TGF-β signaling [40]. It was also suggested that EMT induced by TGF-β1/Snail activation is closely associated with an aggressive growth of CCC.[24] Furthermore, it is known that Slug expression plays a role for the regulation of E-cadherin expression and in the acquisition of invasive potential for human extrahepatic CCC [41]. The known metastatic potential of CCC and the growing role of the Notch signaling pathway in the regulation of bile duct development highlight the necessity to further investigate the mechanisms regulating the migration and invasion of this deadly disease. In order to decipher the role of Notch signaling in regulating CC carcinogenesis *in vitro*, we performed diverse assays. We found that treatment with GSI resulted in an *in vitro* inhibition of migration, invasion, colony formations using wound-healing assay, modified boyden chamber and softagar assay. GSI leads to an increase in the number of cells in the subG1 phase of the cell cycle, which correlates with the increase in the number of apoptotic cells. We also showed a time- and dose-dependent increase of the epithelial marker E-cadherin and of the transcription factor Beta Catenin. The mesenchymal marker Slug and Vimentin an intermediate filament typically found in non-epithelial and mesenchymal cells were both down regulated. In contrast, N-cadherin another mesenchymal marker did not show any changes during GSI treatment. It is important to know that we did not see any significant difference between the two analyzed CC cell lines and that the EMT alteration looks similar under GSI therapy.

p53 is the most commonly mutated tumour suppressor gene associated with the development of human cancer and has been implicated in cholangiocarcinoma development by various studies, including immunohistochemical and molecular epidemiological investigations [42]. It has been previously suggested that p53 alterations in cholangiocarcinoma may be mediated by alterations in intracellular signalling cascades caused by changes in the composition and metabolism of cytotoxic biliary constituents [43]. We hypothesized that overexpression of Notch-ICD together with an inactivation of p53 in a mouse model would accelerate tumor development. In comparison to data from our own working party [32] and a group of control ROSA26/AlbCre mice only, we saw that the additional inactivation of p53 indeed enhanced CC development in our animals, starting with an age of 6 month. Histologic analysis confirmed common features of the carcinoma growing in the mice of our study and human CC. In particular the glandular pattern and changes of ductular reactions in the ductular plate of the maintained portal triads were giving a clue for this assumption. However, mice with an age of 3 month were macro- and microscopically unremarkable.

To conclude, we show for the first time that Notch signaling regulates CC carcinogenesis *in vitro* and that *in vivo* over expression of the intracellular domain of Notch (Notch-ICD) together with an inactivation of the tumor suppressor p53 results in a more severe CC tumor burden comparable to human CCs. Our results strengthen the crucial role of Notch signaling for CC development and the rationale for using GSI as a therapeutic strategy in this deadly disease.

Supporting Information

Figure S1 GSI IX inhibits cell proliferation of EGI 1 cells. The cell proliferation of EGI 1 cells was measured by cell proliferation assay, GSI (5 μM, 20 μM, 40 μM) inhibited cell proliferation in a dose- and time-dependent manner.
(TIF)

Figure S2 GSI IX induces apoptotic cell death in human cholangiocarcinoma cell lines. (A) Annexin V staining detects the percentage of apoptotic cells after GSI IX treatments (5 μM, 20 μM, 40 μM) and DMSO at two different time points (48 and 96 hours). (B) The subG1 phase of the cell cycle was analyzed at 96 hours by FACS in SZ1 and TFK1 cell lines after treatment with GSI IX (5, 10 and 15 μM) and DMSO.
(TIF)

Figure S3 EMT markers are expressed in human cholangiocarcinoma. N- and E-cadherin are highly expressed in representative human cholangiocarcinoma tissues (magnification 20x).
(TIF)

Figure S4 Cytokeratin 7 staining confirmed the epithelial lineage of the tumor cells. Immunohistochemical staining for CK7 for a tumour growing in the liver of a nine month old mouse (NotchIc::AlbCre::p53 L/L). As seen also by H&E staining the tumour cells give evidence for a cholangiolar differentiation (magnification 40×).
(TIF)

Acknowledgments

We are grateful to T. Sudo (Toray Industries Inc., Kamakura, Japan) and Taeko Kobayashi (Institute for Virus Research, Kyoto, Japan) for Hes1 antibody.

Author Contributions

Conceived and designed the experiments: RRP. Performed the experiments: MEK PB VP. Analyzed the data: MEK PB VP LW RRP. Contributed reagents/materials/analysis tools: LW. Wrote the paper: MEK PB VP LW RRP. Critical revision of the manuscript: MEK PB VP NPM LW RRP.

References

1. von Hahn T, Ciesek S, Wegener G, Plentz RR, Weismuller TJ, et al. (2011) Epidemiological trends in incidence and mortality of hepatobiliary cancers in Germany. Scand J Gastroenterol 46: 1092–1098.
2. Shaib Y, El-Serag HB (2004) The epidemiology of cholangiocarcinoma. Semin Liver Dis 24: 115–125.
3. Carriaga MT, Henson DE (1995) Liver, gallbladder, extrahepatic bile ducts, and pancreas. Cancer 75: 171–190.
4. Gatto M, Alvaro D (2010) New insights on cholangiocarcinoma. World J Gastrointest Oncol. 15: 136–145.
5. Welzel TM, McGlynn KA, Hsing AW, O'Brien TR, Pfeiffer RM (2006) Impact of classification of hilar cholangiocarcinomas (Klatskin tumors) on the incidence of intra- and extrahepatic cholangiocarcinoma in the United States. J Natl Cancer Inst 98: 873–875.
6. Claessen MM, Vleggaar FP, Tytgat KM, Siersema PD, van Buuren HR (2009) High lifetime risk of cancer in primary sclerosing cholangitis. J Hepatol 50: 158–164.
7. Tyson GL, El-Serag HB (2011) Risk factors for cholangiocarcinoma. Hepatology 54: 173–184.
8. Khan SA, Taylor-Robinson SD, Davidson BR, Taylor-Robinson SD (2005) Cholangiocarcinoma: seminar. Lancet 366: 1303–1314.
9. Kiesslich T, Wolkersdorfer G, Neureiter D, Salmhofer H, Berr F (2009) Photodynamic therapy for non-resectable perihilar cholangiocarcinoma. Photochem Photobiol Sci 8: 23–30.
10. Falkson G, MacIntyre JM, Moertel CG (1984) Eastern Cooperative Oncology Group experience with chemotherapy for inoperable gallbladder and bile duct cancer. Cancer 54: 965–969.
11. Khan S, Davidson BR, Goldin RD, Heaton N, Karani J, et al. (2012) Guidelines for the diagnosis and treatment of cholangiocarcinoma: an update. GUT 61: 1657–1669.
12. Tannapfel A, Benicke M, Katalinic A, Uhlmann D, Kockerling F, et al. (2000) Frequency of p16(INK4A) alterations and K-ras mutations in intrahepatic cholangiocarcinoma of the liver. Gut 47: 721–727.
13. Tannapfel A, Sommerer F, Benicke M, Katalinic A, Uhlmann D, et al. (2003) Mutations of the BRAF gene in cholangiocarcinoma but not in hepatocellular carcinoma. Gut 52: 706–712.
14. Deshpande V, Nduaguba A, Zimmerman SM, Kehoe SM, Macconaill LE, et al. (2011) Mutational profiling reveals PIK3CA mutations in gallbladder carcinoma. BMC Cancer 11: 60.
15. Hezel AF, Deshpande V, Zhu AX (2010) Genetics of biliary tract cancers and emerging targeted therapies. J Clin Oncol. 28: 3531–3540.
16. Tannapfel A, Weinans L, Geissler F, Schutz A, Katalinic A, et al. (2000) Mutations of p53 tumor suppressor gene, apoptosis, and proliferation in intrahepatic cholangiocellular carcinoma of the liver. Dig Dis Sci 45: 317–324.
17. Khan SA, Thomas HC, Toledano MB, Cox IJ, Taylor-Robinson SD (2005) p53 mutations in human cholangiocarcinoma: a review. Liver Int. 25: 704–716.
18. Thongprasert S, Napapan S, Charoentum C, Moonprakan S (2005) Phase II study of gemcitabine and cisplatin as first-line chemotherapy in inoperable biliary tract carcinoma. Ann Oncol 16: 279–281.
19. Lee GW, Kang JH, Kim HG, Lee JS, Jang JS (2006) Combination chemotherapy with gemcitabine and cisplatin as first-line treatment for immunohistoch emically proven cholangiocarcinoma. Am J Clin Oncol 29: 127–131.
20. Koch U, Radtke F (2010) Notch signaling in solid tumors. Curr Top Dev Biol 92: 411–455.
21. Artavanis-Tsakonas S, Rand MD, Lake RJ (1999) Notch signaling: cell fate control and signal integration in development. Science 284: 770–776.
22. Ishimura N, Bronk SF, Gores GJ (2005) Inducible Nitric Oxide Synthase Up-Regulates Notch-1 in Mouse Cholangiocytes: Implications for Carcinogenesis. Gastroenterology 128: 1354–1368.
23. Geisler F, Nagl F, Mazur PK, Lee M, Zimber-Strobl U, et al. (2008) Liver-specific inactivation of Notch2, but not Notch1, compromises intrahepatic bile duct development in mice. Hepatology 48: 607–616.
24. Lorent K, Yeo SY, Oda T, Chandrasekharappa S, Chitnis A, et al. (2004) Inhibition of Jagged-mediated Notch signaling disrupts zebrafish biliary development and generates multi-organ defects compatible with an Alagille syndrome phenocopy. Development 131: 5753–5766.
25. Ryan MJ, Bales C, Nelson A, Gonzalez DM, Underkoffler L, et al. (2008) Bile duct proliferation in Jag1/fringe heterozygous mice identifies candidate modifiers of the Alagille syndrome hepatic phenotype. Hepatology 48: 1989–1997.
26. Zong Y, Panikkar A, Xu J, Antoniou A, Raynaud P, et al. (2009) Notch signaling controls liver development by regulating biliary differentiation. Development 136: 1727–1739.
27. Yoon HA, Noh MH, Kim BG, Han JS, Jang JS, et al. (2011) Clinicopathological significance of altered Notch signaling in extrahepatic cholangiocarcinoma and gallbladder carcinoma. World J Gastroenterol 17: 4023–4030.
28. Fan B, Malato Y, Calvisi DF, Naqvi S, Razumilava N, et al. (2012) Cholangiocarcinomas can orginate from hepatocytes in mice. J Clin Invest 122: 2911–2915.
29. Sekiya S, Suzuki A (2012) Intrahepatic cholangiocarcinoma can arise from Notch-mediated conversion oh hepatocytes. J Clin Invest 122 : 3914–3918.
30. Dill MT, Tornillo L, Fritzius T, Terracciano L, Semela D, et al. (2013) Constitutive Notch2 signaling induces hepatic tumors in mice. Hepatology 57: 1607–1619.
31. Zhou Q, Wang Y, Peng B, Liang L, Li J (2013) The roles of Notch1 expression in the migration of intrahepatic cholangiocarcinoma. BMC Cancer 13: 244.
32. Zender S, Nickeleit I, Wuestefeld T, Sörensen I, Dauch D, etal. (2013) A critical role for Notch signaling in the formation of cholangiocellular carcinomas. Cancer Cell 23: 783–795.
33. Maeda M, Johnson KR, Wheelock MJ (2005) Cadherin switching: essential for behavioral but not morphological changes during an epithelium-to-mesenchymal transition. J Cell Sci 118: 873–887.
34. Jing Y, Han Z, Zhang S, Liu Y, Wei L (2011) Epithelial-Mesenchymal Transition in tumor microenvironment. Cell Biosci 31: 29.
35. Murtaugh LC, Stanger BZ, Kwan KM, Melton DA (2003) Notch signaling controls multiple steps of pancreatic differentiation. Proc Natl Acad Sci U S A 100: 14920–14925.
36. Kellendonk C, Opherk C, Anlag K, Schütz G, Tronche F (2000) Hepatocyte-specific expression of Cre recombinase. Genesis 26: 151–153.
37. Thiery JP, Sleeman JP (2006) Complex networks orchestrate epithelial–mesenchymal transitions. Nature Reviews Molecular Cell Biology 7: 131–142.
38. Jing Y, Han Z, Zhang S, Liu Y, Wei L (2011) Epithelial-Mesenchymal Transition in tumor microenvironment. Cell Biosci 1: 29.
39. Omenetti A, Porrello A, Jung Y, Yang L, Popov Y, et al. (2008) Hedgehog signaling regulates epithelial-mesenchymal transition during biliary fibrosis in rodents and humans. J Clin Invest 118: 3331–3342.
40. Araki K, Shimura T, Suzuki H, Tsutsumi S, Wada W, et al. (2011) E/N-cadherin switch mediates cancer progression via TGF-beta-induced epithelial-to-mesenchymal transition in extrahepatic cholangiocarcinoma. Br J Cancer 105: 1885–1893.
41. Dormoy V, Danilin S, Lindner V, Thomas L, Rothhut S, et al. (2009) The sonic hedgehog signaling pathway is reactivated in human renal cell carcinoma and plays orchestral role in tumor growth. Mol Cancer 8: 123.
42. Khan SA, Thomas HC, Toledano MB, Cox IJ, Taylor-Robinson SD (2005) p53 Mutations in human cholangiocarcinoma: a review. Liver Int. 25: 704–716.
43. Furubo S, Harada K, Shimonishi T, Katayanagi K, Tsui W, et al. (1999) Protein expression and genetic alterations of p53 and ras in intrahepatic cholangiocarcinoma. Histopathology 35: 230–240.
